# The Association between Specific Substances of Abuse and Subcortical Intracerebral Hemorrhage Versus Ischemic Lacunar Infarction

**DOI:** 10.3389/fneur.2014.00174

**Published:** 2014-09-10

**Authors:** Emma H. Kaplan, Rebecca F. Gottesman, Rafael H. Llinas, Elisabeth B. Marsh

**Affiliations:** ^1^Kennedy-Krieger Institute, Baltimore, MD, USA; ^2^Department of Neurology, Johns Hopkins University School of Medicine, Baltimore, MD, USA; ^3^Department of Neurology, Johns Hopkins Bayview Medical Center, Baltimore, MD, USA

**Keywords:** intracerebral hemorrhage, stroke, hypertension, tobacco, alcohol, drug abuse

## Abstract

**Background:** Hypertension damages small vessels, resulting in both lacunar infarction and subcortical intracerebral hemorrhage (ICH). Substance abuse has also been linked to small vessel pathology. This study explores whether the use of *specific* substances (e.g., cocaine, tobacco) is associated with subcortical ICH over ischemia in hypertensive individuals.

**Methods:** Patients with hypertension, admitted with lacunar infarcts (measuring <2.0 cm) or subcortical ICH, were included in analysis. Brain MRIs and head CTs were retrospectively reviewed along with medical records. Demographic information and history of substance use (illicit/controlled: cocaine, heroin, marijuana, benzodiazepines, and methadone; alcohol; and tobacco) was obtained. “Current use” and “history of use” were determined from patient history or a positive toxicology screen. “Heavy use” was defined as: smoking- ≥0.5 packs per day or 10 pack-years; alcohol- average of >1 drink per day (women), >2 drinks per day (men). Logistic regression was performed with ICH as the dependent variable comparing those presenting with ICH to those presenting with ischemia.

**Results:** Of the 580 patients included in analysis, 217 (37%) presented with ICH. The average age was similar between the two groups (64.7 versus 66.3 years). Illicit/controlled drug use was associated with a significantly increased risk of ICH over stroke in unadjusted models (25 versus 15%, *p* = 0.02), with the largest effect seen in users ≥65 years old (not statistically significant). Smoking was associated with ischemia over ICH in a dose-dependent manner: any history of smoking OR 1.84, CI 1.19–2.84; current use OR 2.23, CI 1.37–3.62; heavy use OR 2.48, CI 1.50–4.13. Alcohol use was not preferentially associated with either outcome (*p* = 0.29).

**Conclusion:** In hypertensive patients, tobacco use is associated with an increased risk of subcortical ischemia compared to ICH, while use of illicit/controlled substances appears to be predictive of hemorrhage.

## Introduction

Hypertension is estimated to affect over a third of adults ≥20 years of age in the United States, but fewer than half maintain adequate blood pressure control (1, 2). Hypertension is a major risk factor for stroke, and 77% of first-time strokes occur in hypertensive patients (2). Lacunar infarcts (lacunes), caused by occlusion of a single small artery in a subcortical brain region, account for about a fifth of all strokes (3). Lacunes tend to have a stronger association with hypertension than non-lacunar, cortical strokes (3). Hypertension is also the leading cause of hemorrhagic stroke, particularly involving the subcortical white matter and deep gray structures (4). The majority of studies addressing the impact of other vascular risk factors combine ischemic stroke, intracerebral hemorrhage (ICH), and subarachnoid hemorrhage without differentiating between stroke subtypes (2, 5). Given that 87% of all strokes are ischemic (2), such data disproportionately represent ischemic infarcts and fail to account for risk factors that may contribute differently to ischemic versus hemorrhagic strokes. Because hypertension is so prevalent and such an important risk factor for both lacunar disease and subcortical ICH (3, 6), the identification of other risk factors preferentially leading to one subtype over the other may be useful in individualizing primary prevention strategies.

The relationship between substance abuse and stroke has been studied, though with little differentiation between stroke subtypes. An estimated 9.2% of adolescents and adults in the United States are current users of illicit/controlled drugs, and an even larger proportion of the population uses legal substances, such as tobacco (22.1%) and alcohol (52.1%) (7). Use of any of these substances has been associated with a higher risk of both ICH and ischemic stroke (8–10).

Our study examines the relationship between substance use and ischemic versus hemorrhagic stroke in hypertensive patients, at higher risk for both. As ICH is typically associated with a poorer long-term prognosis than ischemic stroke (11, 12), a better understanding of the specific factors that increase the risk of hemorrhage over ischemia could aid in potential counseling and specific patient-centered risk modification.

## Materials and Methods

### Study population

The Johns Hopkins University School of Medicine Institutional Review Board approved this study. Patient histories, laboratory results, and imaging studies were reviewed retrospectively for 1,980 individuals presenting to the Johns Hopkins Bayview Medical Center with an ischemic stroke or ICH between 2004 and 2012. The following criteria were required for inclusion in further analysis: age ≥18 years, a “history” of hypertension (defined by reported history, use of hypertensive medications, and/or a finding of left ventricular hypertrophy (LVH) on echocardiogram), and subcortical ICH or lacunar infarct on non-contrast head CT or MRI. Patient histories and laboratory results were reviewed to determine demographic information (age, sex, and race), history of hypertension, and history of substance use/abuse (see below).

### Neuroimaging

Neuroimaging was retrospectively reviewed by two board certified vascular neurologists for the presence of subcortical disease. We have previously demonstrated excellent inter-rater reliability (kappa 0.76) in evaluating ICH (13). MRI was used in the majority of ischemic stroke workups, while non-contrast CT was used (with or without MRI) in most cases of ICH, and when MRI was not possible (e.g., presence of a pacemaker). A 3-T scanner with a standard quadrature transmit–receive head coil was used for all imaging. T1- and T2-weighted imaging was used to evaluate pathology and to determine the age of any hemorrhage seen on susceptibility-weighted imaging (SWI) (14). Diffusion weighted MRI (DWI) was used to visualize acute infarction.

### Defining subcortical lacunes and ICH

A “symptomatic lacune” was defined as a single, focal, DWI/T2-weighted hyperintense lesion measuring <2.0 cm (15) in a classic location for lacunar disease (basal ganglia, cerebellum, pons, medulla, midbrain, or subcortical white matter) that corresponded to the acute neurological presentation. Cases of territorial infarcts, where one or more branches of the lenticulostriate arteries were affected due to occlusion of the middle cerebral artery, resulting in small strokes that appeared lacunar, were excluded.

Presence of subcortical ICH was initially determined using non-contrast CT. To be considered subcortical, a hemorrhage had to originate from the subcortical white matter or deep structures. Cortical hemorrhages, defined as those with only cortical and no deep brain involvement, and hemorrhagic conversion of ischemic stroke, were excluded. When necessary, T1- and T2-weighted MRI imaging was used to confirm the origin of hemorrhage (14).

### Defining substance use/abuse

The use of both legal (alcohol and tobacco) and illicit/controlled (cocaine, heroin, marijuana, benzodiazepines, and methadone) substances was evaluated. “History” and “current use” of any substance was established using toxicology screens and/or patient admission of prior or current use, respectively. Positive toxicology screen results overrode denials in patient history. “Heavy use” of tobacco was defined as ≥0.5 packs per day or 10 pack-years in men and women. “Heavy use” of alcohol in men was defined as an average of >2 drinks per day and in women an average of >1 drink per day (16). “Heavy use” could not be defined in illicit/controlled drug use, as dosage information was unavailable.

### Statistical analysis

Paired *t*-tests (for continuous variables) and Fisher’s exact tests (for categorical variables) were used in initial univariate analysis. Multivariable logistic regression was then performed to adjust for confounding demographic factors including age, sex, and race. In multivariable analysis, ICH (versus ischemia) was the dependent variable, while various forms and degrees of substance use/abuse served as the independent variables. All analyses compared those presenting with subcortical ICH to those presenting with lacunar infarction. As a secondary analysis, groups were stratified by age (less than versus greater than or equal to 65 years), and the effect of intensity of use was explored for each substance.

## Results

Of the 1,980 patients initially screened for inclusion in the study, 580 met criteria after review of neuroimaging. Three hundred sixty-three had subcortical lacunar infarcts and the remaining 217 subcortical ICH. Of those excluded, 63% had cortical pathology and 13% had no visible lesion. The remaining 24% were removed from analysis for other reasons (e.g., a cardioembolic source found during work-up). The average age of the included cohort was 65.6 years. Fifty-four percent were female and 27% were African-American. Baseline demographics of the included cohort did not vary significantly from the entire cohort. Patient characteristics are displayed in Table 1.

**Table 1:** **Patient characteristics**.

Demographics	Total analyzed (*n* = 580)	Lacunar infarct (*n* = 363)	ICH (*n* = 217)	*p*-Value
Age (mean year)	65.6	66.3	64.7	0.28
Sex (% female)	54	53	56	0.48
Race (% African-American)	27	23	34	**0.01**
Tobacco (%)				
History	60.8	64.2	52.9	**0.03**
Current	35.5	39.7	25.9	**0.005**
Heavy	45.6	50.6	33.0	**<0.005**
Alcohol (%)				
History	49.5	47.8	53.3	0.30
Current	41.6	40.5	43.8	0.53
Heavy	16.8	15.9	18.8	0.48
Illicit/controlled substances (%)				
History	18.2	14.8	25.0	**0.02**
Current	14.0	11.8	18.6	0.09

*Bold font indicates statistically significant results*.

### Substances associated with hemorrhage

**Figure 1:**
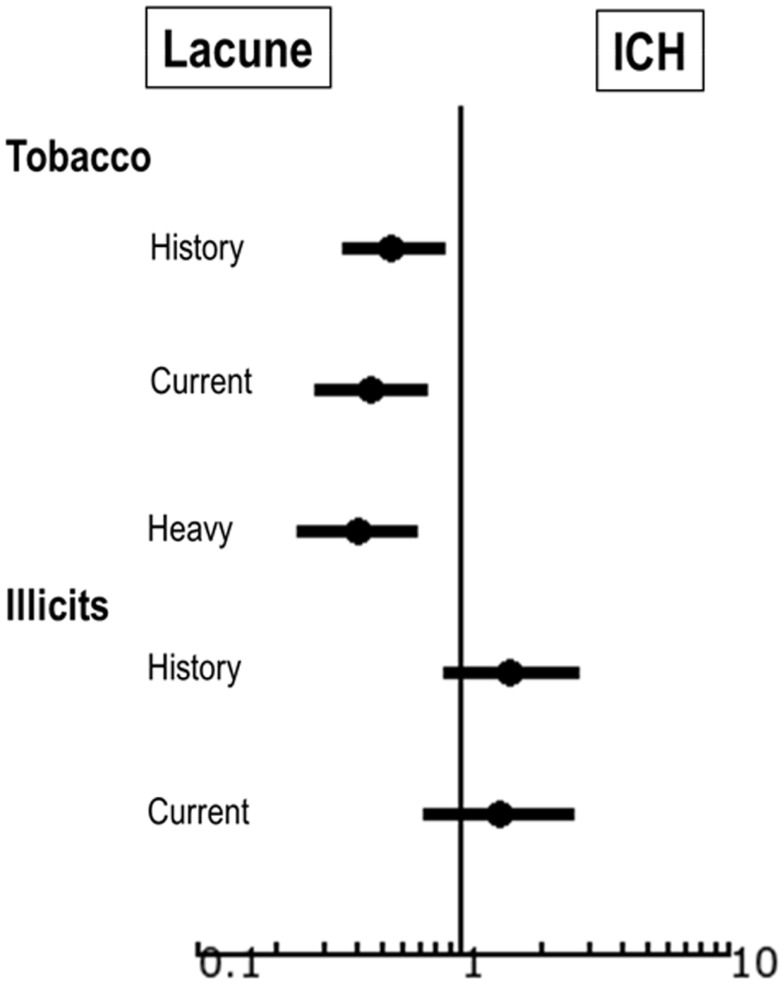
**Forest plot showing factors predictive of subcortical ICH versus lacunar infarct using model adjusted for age, race, and sex**.

Complete results are displayed in Table 1. In univariate analysis, history of illicit/controlled drug use was significantly associated with ICH over ischemia (*p* = 0.02) and current use also trended toward hemorrhage (*p* = 0.09). Smoking was associated with ischemia over ICH (Figure 1). In secondary analysis, the effect appeared dose-dependent: any history of smoking OR 1.84 of smoking over ICH, CI 1.19–2.84; current use OR 2.23 of smoking over ICH, CI 1.37–3.62; heavy use OR 2.48 of smoking over ICH, CI 1.50–4.13. Alcohol was not preferentially associated with either outcome (Figure 1). In multivariate analysis (adjusted for age, race, and sex), in individuals using illicit/controlled drugs the largest effect on increased risk of hemorrhage was seen in individuals age 65 years and greater (OR 1.5 versus 2.5 for current, and 1.7 versus 2.5 for history of use) (Table 2), though results were not statistically significant.

**Table 2:** **Odds ratios for ICH over lacunar infarct stratified by age**.

Use	OR of ICH over lacune	95% Confidence interval
Current
<65 years	1.5	0.77–2.85
≥65 years	2.5	0.48–12.60
History
<65 years	1.7	0.94–3.21
≥65 years	2.5	0.68–8.91

*Adjusted for age, race, and sex*.

## Discussion

Hypertension is prevalent and an important risk factor for both ischemic and hemorrhagic stroke. Specific vascular risk factors may shift the likelihood of one subtype over the other (17). When specifically evaluating substance abuse, tobacco was more strongly associated with ischemia in a dose-dependent manner, while illicit/controlled drug use predicted ICH.

### Tobacco use versus substance abuse – the duration and intensity of abuse

The association between tobacco use and ischemic stroke is not surprising. Cigarette smoking has been linked to both small and large vessel atherosclerotic disease and extensively studied in both the cerebrovascular and cardiovascular literature (10, 18, 19). The relationship between illicit/controlled drug use and hemorrhage is somewhat less intuitive. We posit several potential mechanisms. First and foremost, the population may be different. Individuals who abuse illicit/controlled substances have higher rates of hepatitis C and other chronic illnesses (20), which may result in a relative coagulopathy. On further review of our data, there did not seem to be major differences in the demographic profile between those with hemorrhage versus infarction (17), but we were unable to adjust for all possible medical co-morbidities, including liver disease. A second possibility is that “street drugs” are often cut with agents that may be caustic to blood vessels and result in increased permeability. Furthermore, the use of cocaine results in short, rapid elevations of blood pressure that may cause relatively leaky vessels to rupture, resulting in hemorrhage. While all of these theories may contribute to increased hemorrhage risk, we propose an alternative mechanism related to intensity and duration of use. Those who smoke cigarettes tend to do so consistently (multiple times a day on a daily basis); whereas the use of illicit/controlled substances is typically more episodic, done in a “binge-like” fashion. Continuous damage to vessels through cigarette smoking over time results in thickening of the arteries and plaque build-up, decreasing the diameter of small vessels, and increasing the risk for small vessel ischemia. Due to cost and difficulty of procurement, illicit/controlled substances are often used sporadically. Rather than gradually hardening the vessel wall through consistent repetitive damage, this results in vessel injury without thickening or rigidity. In these cases, severe hypertension is more likely to result in friable vessel rupture and subsequent hemorrhage.

The concept that intensity and duration of exposure to a risk factor directly impacts the resulting stroke subtype is consistent with the dose–response relationship we observed with tobacco abuse. Heavier, more frequent use amplifies vessel narrowing without rupture. We have described a similar phenomenon with other vascular risk factors, also dependent on intensity and duration (17). In this study, we also found that the effect of drug use on predicting ICH over ischemia tended to be higher in individuals over the age of 65, though results were not statistically significant. This may be due to longer exposure to other chronic risk factors, such as hypertension, resulting in vessels with significantly abnormal pathology increasing the risk for both stroke subtypes. When exposed to an acute injury in the setting of illicit/controlled drug use, these friable vessels may be even more prone to rupture.

### Effect of alcohol use

Surprisingly, alcohol use was not preferentially associated with either ischemia or hemorrhage. There are several potential explanations. Studies have shown that moderate levels of alcohol intake increase risk of stroke and cardiac disease, while lower levels appear to be protective (21). This is consistent with our hypothesis that chronic, consistent, moderate to heavy substance use thickens vessel walls over time making thrombosis (ischemia) more likely than rupture (ICH). We did not observe this effect with alcohol in our population. However, we compared ischemia to ICH rather than a control group. Patients who chronically use alcohol are also often malnourished and have relative coagulopathies related to underlying liver disease and/or platelet abnormalities. A co-existent increased risk for hemorrhage may have washed out the effect of increased risk of ischemia. An additional potential explanation is that, given the retrospective nature of the study, alcohol use was poorly recorded. In our experience, compared to both smoking and illicit/controlled drug use, it is a more socially acceptable substance of abuse and often patients will underestimate their true alcohol intake. We relied on patient reports without the ability for further clarification both into overall use and degree of intake. This may have affected the results.

### Limitations

Our study is not without limitations. It is a retrospective analysis of prospectively collected data, preventing further quantification of illicit/controlled drug use. It is also impossible to ensure that all patients were adequately screened for substance use history, particularly those presenting with aphasia or altered mental status, though all patients admitted to our institution typically undergo screening for substance use as part of their social history. When a patient is unable to provide the history themselves, if available, a family member is asked to provide the information. Additionally, it includes a relatively small number of patients from a single institution. We may therefore be underpowered to show an effect of alcohol or current drug use, or alternatively may have a higher rate of substance use/abuse and underlying co-morbidities than other populations. To that end, there are likely other unmeasured confounders that we were unable to adequately account for, such as hepatitis, which may affect clotting factors. Finally, it is a cross-sectional analysis rather than a longitudinal study. An association between substance use and stroke subtype does not necessarily indicate a causal pathway; however, such an association may indicate a potential mechanism that can be further studied. Even with these limitations, our data strongly suggest that the type and degree of substance use increases the risk for certain stroke subtypes.

## Conclusion

In hypertensive patients, tobacco use is highly associated with an increased risk of subcortical ischemia compared to ICH in a dose-dependent manner, while use of illicit/controlled substances appears to be predictive of subcortical ICH.

## Conflict of Interest Statement

The authors declare that the research was conducted in the absence of any commercial or financial relationships that could be construed as a potential conflict of interest.
